# Contrasting Outcomes in Chronic Monteggia Fracture Management: Key Lessons From Two Pediatric Cases

**DOI:** 10.7759/cureus.102592

**Published:** 2026-01-29

**Authors:** Nazrin Radzuan, Imma Isniza Ismail, Norazian Kamisan

**Affiliations:** 1 Pediatric Orthopedic Unit, Universiti Putra Malaysia, Serdang, MYS

**Keywords:** chronic radial head dislocation, monteggia fracture, pediatric elbow trauma, sliding oblique osteotomy, ulnar osteotomy

## Abstract

Chronic Monteggia fracture-dislocations in children, defined by presentation beyond four weeks, pose significant surgical challenges. While ulnar osteotomy is central to reconstruction, the optimal technique for ensuring stable radiocapitellar reduction remains a topic of debate. We present the cases of two nine-year-old patients with chronic Bado type I Monteggia lesions. The first patient underwent transverse ulnar osteotomy with annular ligament repair and temporary radiocapitellar fixation but developed recurrent radial head dislocation after K-wire removal. The second patient was treated with a sliding oblique ulnar osteotomy, achieving stable reduction without recurrence and maintaining full elbow motion. These contrasting outcomes underscore the importance of osteotomy configuration. Transverse osteotomies, although simple, offer limited bone contact and controlled correction, which can compromise stability. In contrast, the sliding oblique osteotomy provides increased cortical contact area and intrinsic stability, allowing for precise lengthening and angulation, which is critical in the setting of chronic soft-tissue contracture and joint incongruity. In the surgical management of chronic pediatric Monteggia lesions, the choice of ulnar osteotomy significantly impacts stability. A sliding oblique ulnar osteotomy offers biomechanical advantages over a transverse osteotomy, including greater inherent stability and controlled deformity correction, which may reduce the risk of recurrent dislocation. Surgical planning should prioritize osteotomy geometry to optimize outcomes.

## Introduction

Chronic Monteggia fracture-dislocations represent a challenging subset of pediatric elbow injuries characterized by persistent radial head dislocation associated with ulnar deformity presenting more than four weeks after injury [1]. These injuries are frequently missed during initial assessment, particularly when ulnar plastic deformation is subtle, leading to delayed diagnosis and complex reconstructive challenges [2]. Prolonged dislocation results in adaptive changes, including annular ligament insufficiency, capsular contracture, and remodeling of the radial head and capitellum, all of which compromise joint stability [3].

Surgical management is generally indicated in chronic cases to restore alignment and function. Ulnar osteotomy has been widely recognized as the cornerstone of treatment, as restoration of ulnar length and alignment facilitates indirect reduction and stabilization of the radial head through tensioning of the interosseous membrane [2,3]. However, controversy persists regarding the optimal osteotomy configuration, particularly in chronic cases where maintaining reduction remains difficult [1]. This case series presents two pediatric patients with chronic Bado type I Monteggia lesions treated using different ulnar osteotomy techniques, highlighting the influence of osteotomy geometry on surgical outcome.

## Case presentation

Case 1

**Figure 1:**
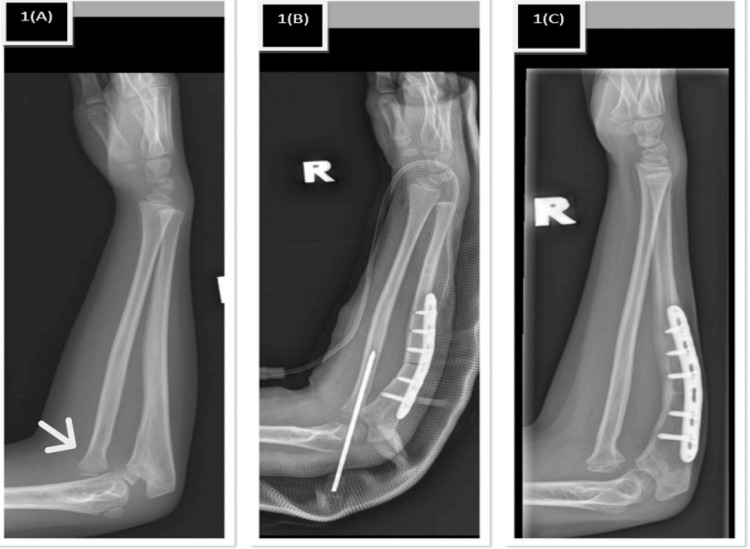
Plain radiograph of patient 1. (1A) Bado type 1 with plastic deformation of the ulna. (1B) Post-ulna transverse osteotomy with transcapitellar K-wire fixation of the radial head. (1C) Anterior radial head redislocation after K-wire removal with united ulnar osteotomy.

A nine-year-old right-hand-dominant girl presented with a three-year history of progressive right elbow deformity following a fall, with the initial injury missed at first presentation. Clinical examination revealed cubitus valgus deformity of approximately 25°, with preserved elbow motion including flexion-extension from 0° to 140° and full forearm rotation. Radiographs confirmed a chronic Bado type I Monteggia lesion with plastic deformation of the ulna (Figure 1).

The patient underwent open reduction and acute ulnar lengthening via a transverse osteotomy at the metadiaphyseal region. Intraoperatively, fibrous tissue interposed within the radiocapitellar joint was excised, and the radial head was reduced and stabilized using a transcapitellar Kirschner wire, with concurrent annular ligament repair performed. The radial head epiphysis and capitellum were noted to be dysplastic and incongruent, consistent with long-standing dislocation.

Early postoperative recovery was uneventful; however, recurrent anterior radial head dislocation was noted six weeks postoperatively following K-wire removal, without any intervening trauma (Figure 1). Elbow range of motion remained unchanged from the preoperative state, and residual deformity persisted, reflecting loss of radiocapitellar stability.

Case 2

**Figure 2:**
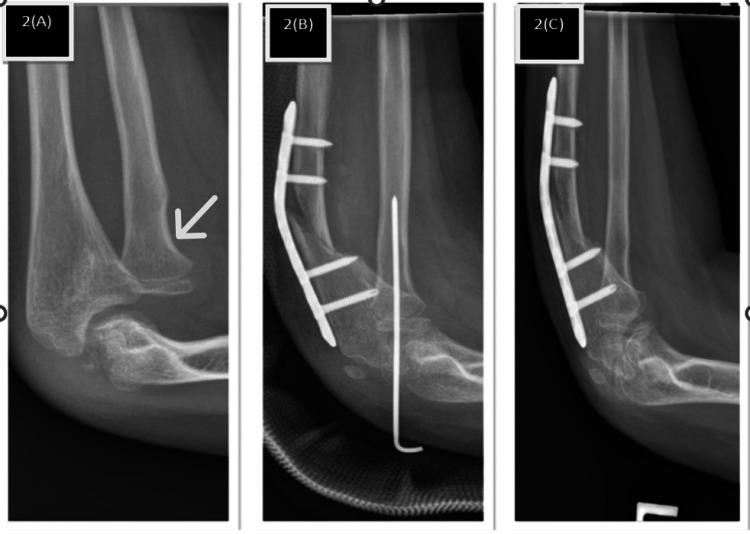
Plain radiograph of patient 2. (2A) Bado type 1 with no obvious deformity of the ulna. (2B) Post-ulna sliding osteotomy with transcapitellar K-wire fixation of the radial head. (2C) The radial head remained reduced after K-wire removal with united ulnar osteotomy.

A nine-year-old boy presented with a one-year history of left elbow deformity following a childhood fracture initially treated non-operatively. Examination demonstrated cubitus valgus deformity of approximately 18°, with preserved full elbow motion and no neurological deficit. Radiographs showed a chronic Bado type I Monteggia lesion with minimal apparent ulnar deformity (Figure 2). Surgical intervention was performed one year after the initial injury and consisted of a sliding oblique ulnar osteotomy with approximately 7 mm of controlled lengthening in the sagittal plane. Radial head reduction was achieved and stabilized with a transcapitellar K-wire, which was maintained for six weeks. Postoperative radiographs confirmed maintained radiocapitellar alignment, and the patient retained full preoperative range of motion without evidence of redislocation (Figure 2).

## Discussion

The management of chronic Monteggia fracture-dislocations in pediatric patients remains challenging because long-standing radial head dislocation leads to soft-tissue contracture, annular ligament insufficiency, capsular scarring, and remodeling of the radial head and capitellum, with altered elbow biomechanics [1,2]. These adaptive changes, combined with an often subtle, initial ulnar deformity and a missed diagnosis, make late reconstruction technically demanding and make maintaining a stable radiocapitellar reduction particularly challenging [3,4]. Classification commonly used in Monteggia fracture is Bado's classification. It divides Monteggia fractures into four types based on the direction of radial head dislocation and ulnar fracture angulation, with Type I (anterior dislocation/anterior angulation), Type II (posterior dislocation/posterior angulation), Type III (lateral dislocation) and Type IV (anterior dislocation with radial and ulnar shaft fractures).

Restoration of ulnar length and alignment through ulnar osteotomy is widely regarded as the cornerstone of surgical treatment in chronic Monteggia lesions, as correction of the ulna indirectly facilitates radial head reduction by re-tensioning the interosseous membrane and proximal forearm soft tissues [1,5]. However, the optimal configuration of ulnar osteotomy remains debated, especially in chronic cases where deformity is complex and soft-tissue support is compromised [2]. Traditional transverse osteotomies are technically straightforward and familiar to most surgeons but may offer limited intrinsic stability and less capacity for controlled, multiplanar correction [6].

In the first case, a transverse ulnar osteotomy with acute lengthening, annular ligament repair, and transcapitellar K-wire fixation initially achieved radiocapitellar reduction but was followed by recurrent dislocation after wire removal at six weeks. Several factors likely contributed to this failure, including extensive intraoperative fibrosis, severely torn annular ligament, and dysplastic, incongruent articular surfaces, all of which reduced the ability of the soft tissues and bony anatomy to maintain reduction once temporary fixation was withdrawn [1,3]. The transverse configuration itself provides a relatively small contact area perpendicular to the ulna's long axis, offering less inherent mechanical stability and limited opportunity to fine-tune angulation and length simultaneously in the setting of complex deformity [7].

By contrast, the second case demonstrated a favorable outcome using an oblique sliding ulnar osteotomy with controlled lengthening and transcapitellar K-wire fixation despite the presence of ligamentous laxity and radial head hypoplasia. The oblique orientation increases cortical contact, enhances intrinsic stability, and allows coupled correction of angulation and length in a single construct, which helps restore the proximal ulna’s alignment while providing sustained tension across the interosseous membrane and radiocapitellar joint [1,3]. This configuration offers improved resistance to shear forces and reduced micromotion at the osteotomy site, potentially lowering the risk of loss of correction and recurrent dislocation during the healing phase [1,5].

From a biomechanical standpoint, both techniques aim to re-establish appropriate ulnar alignment and length to indirectly stabilize the radial head, but the oblique sliding osteotomy appears better suited to chronic cases in which multidirectional deformity and soft-tissue compromise are common [2]. The contrasting outcomes in these two patients underscore how osteotomy geometry, along with soft-tissue quality and articular morphology, strongly influences the ability to maintain a stable reduction after temporary fixation is removed [2,3]. In situations with severe annular ligament insufficiency, hypoplastic radial head, or marked capsular contracture, prolonged radiocapitellar fixation beyond six weeks may be prudent to allow soft-tissue adaptation and remodeling, particularly when a less inherently stable osteotomy configuration is used [8].

These cases also highlight the importance of meticulous preoperative planning and individualized intraoperative decision-making in the management of chronic pediatric Monteggia injuries. Preoperative magnetic resonance imaging (MRI) may help define the extent of annular ligament damage, capsular contracture, and radial-capitellar dysplasia, thereby guiding the need for ligament reconstruction, the choice of osteotomy configuration, and the anticipated duration of fixation [1,3]. Ultimately, a tailored approach that prioritizes correction of ulnar deformity, optimizes osteotomy stability, respects soft-tissue biology, and calibrates the length of radiocapitellar fixation is essential to reduce the risk of recurrent dislocation and optimize functional outcomes in this complex pediatric population [2,3]. Further evaluation with a meta-analysis and systematic review, using data derived from cases like this, may contribute to the recommended approach in chronic Monteggia proximal ulna osteotomy in the future.

## Conclusions

Chronic Monteggia fracture-dislocations in pediatric patients require meticulous surgical planning due to the biomechanical and soft-tissue adaptations associated with long-standing radial head dislocation. Restoration of ulnar length and alignment through ulnar osteotomy remains fundamental to achieving stable radiocapitellar reduction. This case series demonstrates that ulnar osteotomy configuration is a key determinant of stability in chronic cases. While transverse ulnar osteotomy remains a technically straightforward option, its limited surface contact and reduced ability to provide controlled angulation may compromise stability in the presence of soft-tissue insufficiency. In contrast, sliding oblique ulnar osteotomy offers superior biomechanical stability through increased cortical contact and precise deformity correction, potentially reducing the risk of recurrent radial head dislocation in chronic pediatric Monteggia fractures. A tailored surgical approach that prioritizes osteotomy geometry, intraoperative stability assessment, and appropriate fixation duration is essential to optimize outcomes in these complex injuries.
